# Supplementary material for: Mitogenomics of five Olidiana leafhoppers (Hemiptera: Cicadellidae: Coelidiinae) and their phylogenetic implications
Source: PeerJ. 2021 Apr 27;9:e11086. doi: 10.7717/peerj.11086 (PMC8086571; doi:10.7717/peerj.11086)
Supplement: Supplemental Information 6 [file peerj-09-11086-s006.doc]

Supplementary Tables

**Table S1. Collection information of specimens.**

| **Number** | **Species** | **Sample site** | **Longitude and latitude** | **Altitude (m)** |
| --- | --- | --- | --- | --- |
| COW9043 | *Olidina alata* | Wenbi Mountain, Xinping County, and Yunnan Province | 101°57′57″E  24°07′39″N | 1,908 |
| COW9065 | *Olidina longsticka* | Dawei Mountain, Pingbian County, and Yunnan Province | 103°13′33″E  22°51′50″N | 1,947 |
| COW9071 | *Olidina obliquea* | Fenshui Mountain, Xinping County, and Yunnan Province | 103°13′33″E  22°51′50″N | 1,809 |
| COW9001X | *Olidina ritcheri* | Wenbi Mountain, Xinping County, and Yunnan Province | 101°57′57″E  24°07′39″N | 1,908 |
| COW9002X | *Olidina tongmaiensis* | Tongmai town, Bowoxian County, and Tibet Province | 96°28′58″E  30°54′42″N | 4,085 |

**Table S2.** Taxonomic information of the species used in this study and their GenBank accession numbers.

| **Subfamily** | **Species** | **GeneBank** | **Length (bp)** | **Reference** |
| --- | --- | --- | --- | --- |
| Cicadellinae | *Bothrogonia ferruginea* | KU167550 | 15,262 | Unpublished |
|  | *Cicadella viridis* **^1^** | KY752061 | 13,461 | Unpublished |
|  | *Homalodisca coagulata* **^1^** | AY875213 | 15,304 | Unpublished |
| Coelidiinae | *Olidiana alata* | MN780581 | 15,205 | **This study** |
|  | *Olidiana longsticka* | MN780582 | 15,993 | **This study** |
|  | *Olidiana olbliquea* | MN780583 | 15,312 | **This study** |
|  | *Olidiana ritcheri* | MN780584 | 15,372 | **This study** |
|  | *Olidiana ritcheriina* | MK738125 | 15,166 | (Wang et al., 2019c) |
|  | *Olidiana* sp. **^1^** | KY039119 | 15,253 | Unpublished |
|  | *Olidiana tongmaiensis* | MN780585 | 15,363 | **This study** |
|  | *Taharana fasciana* | NC036015 | 15,161 | (Wang et al., 2017) |
| Deltocephalinae | *Alobaldia tobae* | KY039116 | 16,026 | Unpublished |
|  | *Abrus expansivus* | MK033020 | 15,904 | (Wang & Xing, 2017) |
|  | *Drabescus ineffectus* | NC050258 | 15,744 | (Xu et al., 2020) |
|  | *Elymana sulphurella* | MK251106 | 15,674 | (Song et al., 2019) |
|  | *Exitianus indicus* | KY039128 | 16,089 | (Song et al., 2017) |
|  | *Hengchunia truncata* | MK251146 | 15,749 | (Song et al., 2019) |
|  | *Hishimonoides recurvatis* | KY364883 | 14,814 | (Song et al., 2018) |
|  | *Japananus hyalinus* | NC036298 | 15,364 | (Du et al., 2017b) |
|  | *Macrosteles quadrimaculatus* | NC039560 | 15,734 | (Du et al., 2019) |
|  | *Maiestas dorsalis* | NC036296 | 15,352 | (Du et al., 2017b) |
|  | *Nephotettix cincticeps* | NC026977 | 14,805 | (Song et al., 2017) |
|  | *Orosius orientalis* | KY039146 | 15,513 | (Song et al., 2017) |
|  | *Paralaevicephalus gracilipenis* | MK450366 | 16,114 | (Xing et al., 2019) |
|  | *Paramacrosteles nigromaculatus* | NC045270 | 15,011 | (Yang et al., 2019) |
|  | *Pellucidus guizhouensis* | MF784429 | 16,555 | Unpublished |
|  | *Reticuluma hamata* | MN922303 | 15,190 | (Xu & Dai, 2020) |
|  | *Roxasellana stellata* | NC050257 | 15,361 | (Xu et al., 2020) |
|  | *Scaphoideus nigrivalveus* | KY817244 | 15,235 | (Du et al., 2017a) |
|  | *Sorhoanus xanthoneurus* | MK251116 | 15,925 | (Song et al., 2019) |
|  | *Stirellus bicolor* | MK251122 | 15,529 | (Song et al., 2019) |
|  | *Yanocephalus yanonis* | NC036131 | 15,623 | (Song et al., 2017) |
| Evacanthinae | *Exitianus indicus* | KY039128 | 16,089 | (Song et al., 2017) |
| Hylicinae | *Balala fujiana* | MW218661 | 16,221 | (Tang et al., 2020) |
|  | *Hylica paradoxa* | MW218660 | 14,762 | (Tang et al., 2020) |
|  | *Kalasha nativa* | MW218662 | 15,716 | (Tang et al., 2020) |
|  | *Nacolus tuberculatus* | MW218663 | 15,737 | (Tang et al., 2020) |
| Iassinae | *Batracomorphus lateprocessu* | MG813489 | 15,356 | (Wang et al., 2020) |
|  | *Gessius rufidorsus* | MN577633 | 14,634 | (Wang et al., 2020) |
|  | *Iassus dorsalis* | MN577634 | 15,176 | (Wang et al., 2020) |
|  | *Krisna concava* | MN577635 | 14,305 | (Wang et al., 2020) |
|  | *Trocnadella arisana* | NC036480 | 15,131 | (Wang et al., 2020) |
| Idiocerinae | *Idioscopus clypealis* | NC039642 | 15,393 | (Dai et al., 2018) |
|  | *Idioscopus myrica* | MH492317 | 15,423 | (Wang et al., 2018) |
|  | *Liocratus salicis* | MG813490 | 16,436 | (Wang et al., 2018) |
|  | [*Populicerus confusus*](https://www.ncbi.nlm.nih.gov/nuccore/NC_039427.1) | NC039427 | 16,494 | (Wang et al., 2018) |
|  | *Rhytidodus viridiflavus* | MN935488 | 16,842 | (Di et al., 2020) |
| Ledrinae | *Ledra auditura* | MK387845 | 16,094 | (Wang et al., 2019a) |
|  | *Petalocephala chlorophana* | NC051527 | 14,927 | (Huang & Zhang, 2020) |
|  | *Tituria pyramidata* | NC046701 | 15,331 | (Li & Dai, 2020) |
|  | *Tituria sagittata* | NC051528 | 14,918 | (Huang & Zhang, 2020) |
| Macropsinae | *Macropsis notata* | NC042723 | 16,323 | (Wang et al., 2020b) |
|  | *Oncopsis nigrofasciata* | MG813492 | 15,927 | (Wang et al., 2020b) |
| Megophthalminae | *Japanagallia spinosa* | NC035685 | 15,655 | (Wang et al., 2017) |
|  | *Durgades nigropicta* | NC035684 | 15,974 | (Wang et al., 2017) |
| Mileewinae | *Mileewa margheritae* | MT483998 | 15,375 | (He & Yang, 2020a) |
|  | *Mileewa ponta* | MT497465 | 15,999 | (He & Yang, 2020b) |
| Typhlocybinae | *Draeculacephala crassicornis* | MK251091 | 15,214 | (Song et al., 2019) |
|  | *Empoasca vitis* | NC024838 | 15,154 | (Liu et al., 2016) |
|  | *Empoascanara sipra* | NC048516 | 14,827 | (Tan et al., 2020) |
|  | *Eratoneura flexibilis* **^1^** | MK251094 | 15,543 | (Song et al., 2019) |
|  | *Eupteryx minuscula* | MN910279 | 16,944 | (Yang et al., 2020) |
|  | *Ghauriana sinensis* | MN699874 | 15,491 | (Shi et al., 2020) |
|  | *Kybos pura* | MK251088 | 14,990 | (Song et al., 2019) |
|  | *Limassolla lingchuanensis* | NC046037 | 15,716 | (Yuan et al., 2020a) |
|  | *Mitjaevia protuberanta* | NC047465 | 15,472 | (Yuan et al., 2020a) |
|  | *Paraahimia luodianensis* | NC047464 | 16,497 | (Song et al., 2020) |
|  | *Parathailocyba orla* | MN894531 | 15,382 | (Jiang et al., 2020) |
|  | *Parazyginella tiani* | MT683891 | 17,562 | (Zhou et al., 2020) |
|  | *Zyginella minuta* | MT488436 | 15,544 | (Zhou et al., 2020) |
| Treehopper | *Centrotus cornutus* **^1^** | KX437728 | 14,696 | (Song et al., 2018) |
|  | *Entylia carinata* | NC033539 | 15,662 | (Mao et al., 2016) |
|  | *Hypsauchenia hardwickii* | NC044705 | 15,618 | (Hu et al., 2019) |
|  | *Maurya qinlingensis* | NC044706 | 16,011 | (Hu et al., 2019) |
|  | *Leptobelus gazella* | NC023219 | 16,007 | Zhao & Liang, 2016) |
|  | *Leptocentrus albolineatus* | NC044707 | 15,508 | (Hu et al., 2019) |
|  | *Tricentrus brunneus* | NC044708 | 16,467 | (Hu et al., 2019) |
| Outgroup | *Cosmoscarta bispecularis* | KP064511 | 15,426 | (Yang et al., 2014) |
|  | *Tettigades auropilosa* **^1^** | KM000129 | 14,944 | Unpublished |

^1^ Incomplete mitogenome sequence

**References**

**Dai RH, Wang JJ, Yang MF. 2018.** The complete mitochondrial genome of the leafhopper *Idioscopus clypealis* (Hemiptera: Cicadellidae: Idiocerinae). *Mitochondrial DNA Part B* **3**:32-33.

**Di XC, Shan LCY, Luo H, Zhang B. 2020.** Complete mitochondrial genome of *Rhytidodus viridiflavus* (Hemiptera: Cicadellidae: Idiocerinae). *Mitochondrial DNA Part B* **5** (2) 1321-1322.

**Du Y, Zhang C, Dietrich CH, Zhang Y, Dai W. 2017b.** Characterization of the complete mitochondrial genomes of *Maiestas dorsalis* and *Japananus hyalinus* (Hemiptera: Cicadellidae) and comparison with other Membracoidea. *Sci*. *Rep*. **7**:14197.

**Du YM, Dietrich CH, Dai W. 2019.** Complete mitochondrial genome of *Macrosteles quadrimaculatus* (Matsumura) (Hemiptera: Cicadellidae: Deltocephalinae) with a shared tRNA rearrangement and its phylogenetic implications. *Int*. *J*. *Biol*. *Macromol*. **122**:1027-1034.

**Du Y, Dai W, Dietrich CH. 2017a.** Mitochondrial genomic variation and phylogenetic relationships of three groups in the genus Scaphoideus (Hemiptera: Cicadellidae: Deltocephalinae). *Sci*. *Rep*. **7**:14197.

**He HL, Yang MF. 2020a.** The mitogenome of *Mileewa margheritae* (Hemiptera: Cicadellidae: Mileewinae). *Mitochondrial DNA Part B* **5** (3),3163-3164.

**He HL, Yang MF. 2020b.** Characterization and phylogenetic analysis of the mitochondrial genome of *Mileewa ponta* (Hemiptera: Cicadellidae: Mileewinae). *Mitochondrial DNA Part B* **5** (3),2976-2977.

**Hu K, Yuan F, Dietrich CH, Yuan XQ. 2019.** Structural features and phylogenetic implications of four new mitogenomes of Centrotinae (Hemiptera: Membracidae). *Int. J. Biol. Macromol.* 139, 1018-1027.

**Huang WJ, Zhang YL. 2020.** Characterization of Two Complete Mitochondrial Genomes of Ledrinae (Hemiptera: Cicadellidae) and Phylogenetic Analysis. *Insects* **11** (9), E609.

**Jiang J, Yuan X, Yuan Z, Song Y. 2020.** The complete mitochondrial genome of *Parathailocyba orla* (Hemiptera: Cicadellidae: Typhlocybinae). *Mitochondrial DNA Part B* **5**(2), 1981-1982.

**Li DF , Dai RH. 2020.** The complete mitochondrial genome of *Tituria pyramidata* (Hemiptera: Cicadellidae: Ledrinae) from China. *Mitochondrial DNA Part B* **5** (2), 1757-1758.

**Li H, Leavengood JM, Chapman EG, Burkhardt D, Song F, Jiang P, Liu JP, Cai WZ. 2017.** Mitochondrial phylogenomics of Hemiptera reveals adaptive innovations driving the diversification of true bugs. *Proc*. *Biol*. *Sci*. **284**:20171223.

**Mao M, Yang X, Bennett G. 2016.** The complete mitochondrial genome of *Entylia carinata* (Hemiptera: Membracidae). *Mitochondrial DNA Part B* **1**:662-663.

**Shi R, Yu XF, Yang MF. 2020.** Complete mitochondrial genome of *Ghauriana sinensis* (Hemiptera: Cicadellidae: Typhlocybinae). *Mitochondrial DNA Part B* **5**(2), 1367-1368.

**Song N, Cai WZ, Li H. 2017.** Deep-level phylogeny of Cicadomorpha inferred from mitochondrial genomes sequenced by NGS. *Sci*. *Rep*. **7**:1-11.

**Song N, Cai W, Li H. 2018.** Insufficient power of mitogenomic data in resolving the auchenorrhynchan monophyly. *Zool*. *J*. *Linn*. *Soc-Lond*. **183**:776-790.

**Song N, Zhang H, Zhao T. 2019.** Insights into the phylogeny of Hemiptera from increased mitogenomic taxon sampling. *Mol*. *Phylogen*. *Evol*. **137**:236-249.

**Song Y, Yuan X, Li C. 2020.** The mitochondrial genome of *Paraahimia luodianensis* (Hemiptera: Cicadellidae: Typhlocybinae), a new genus and species from China. *Mitochondrial DNA Part B* **5**(2), 1351-1352.

**Tan C, Chen X, Li C, Song Y. 2020a.** The complete mitochondrial genome of Empoascanara sipra (Hemiptera:Cicadellidae:Typhlocybinae) with phylogenetic consideration. *Mitochondrial DNA Part B* **5** (1), 260-261.

**Tang J, Huang WJ, Zhang YL. 2020.** The Complete Mitochondrial Genome of Four Hylicinae (Hemiptera: Cicadellidae): Structural Features and Phylogenetic Implications. *Insects* **11**(12).

**Wang JJ, Dai RH, Li H, Zhan HP. 2017.** Characterization of the complete mitochondrial genome of *Japanagallia spinosa* and *Durgades nigropicta* (Hemiptera: Cicadellidae: Megophthalminae). *Biochem*. *Syst*. *Ecol*. **74**:33-41.

**Wang JJ, Li DF, Li H, Yang MF, Dai RH. 2019a.** Structural and phylogenetic implications of the complete mitochondrial genome of *Ledra auditura*. *Sci*. *Rep*. **9**:1-11.

**Wang JJ, Li H, Dai RH. 2017.** Complete mitochondrial genome of *Taharana fasciana* (Insecta, Hemiptera: Cicadellidae) and comparison with other Cicadellidae insects. *Genetica*. **145**:593-602.

**Wang JJ, Wu YF, Dai RH, Yang MF. 2019a.** Comparative mitogenomes of six species in the subfamily Iassinae (Hemiptera: Cicadellidae) and phylogenetic analysis. *Int*. *J*. *Biol*. *Macromol*. **149**:1294-1303.

**Wang JJ, Wu YF, Yang MF. Dai RH. 2020b.** The phylogenetic implications of the mitochondrial genomes of *macropsis notata* and *oncopsis nigrofasciata*. *Front*. *Genet*. **11**:443.

**Wang JJ, Yang MF, Dai RH, Li H. 2019b.** Complete mitochondrial genome of *Evacanthus heimianus* (Hemiptera: Cicadellidae: Evacanthinae) from China. *Mitochondrial DNA Part B* **4**: 284-285.

**Wang JJ, Yang MF, Dai RH, Wang XY. 2018.** Characterization and phylogenetic implications of the complete mitochondrial genome of Idiocerinae (Hemiptera: Cicadellidae). *Int*. *J*. *Biol*. *Macromol*. 120:2366-2372.

**Wang XY, Wang JJ, Fan ZH, Dai RH. 2019c.** Complete mitogenome of *Olidiana ritcheriina* (Hemiptera: Cicadellidae) and phylogeny of Cicadellidae. *PeerJ* **7**:e8072.

**Xing JC, Wang JJ. 2019.** Complete mitochondrial genome of *Abrus expansivus* (Hemiptera: Cicadellidae: Deltocephalinae) from China[J]. *Mitochondrial DNA Part B* **4** (1), 1372-1373.

**Xu D, Yu T, Zhang Y. 2020.** Characterization of the Complete Mitochondrial Genome of *Drabescus ineffectus* and *Roxasellana stellata* (Hemiptera: Cicadellidae: Deltocephalinae: Drabescini) and Their Phylogenetic Implications. *Insects* **11** (8), E534.

**Xu TL, Dai RH. 2020.** Complete mitogenome of *Reticuluma hamata* (Hemiptera: Cicadellidae: Deltocephalinae) from China. *Mitochondrial DNA Part B* **5** (2), 1437-1438.

**Yang H, Liu J, Liang AP. 2014.** The complete mitochondrial genome of *Cosmoscarata bispecularis* (Hemiptera, Cicadomorpha, Cercopoidea, Cercopidae). *DNA Seq*. **27**:3957-3958.

**Yang W, Gao Y, Li C, Song Y. 2019.** The complete mitochondrial genome of *Chlorotettix nigromaculatus* (Hemiptera: Cicadellidae: Deltocephalinae) with phylogenetic consideration. *Mitochondrial DNA Part B* **4** (1), 624-625.

**Yang XS, Mao M, Bennett G. 2017.** The complete mitochondrial genome of *Macrosteles quadrilineatus* (Hemiptera: Cicadellidae). *Mitochondrial DNA Part B* **2**:173-175.

**Yang X, Yuan Z, Li C, Song,Y. 2020b.** Complete mitochondrial genome of *Eupteryx*(Stacla) *minusula* (Hemiptera: Cicadellidae: Typhlocybinae) from China. *Mitochondrial DNA Part B* **5** (3), 2375-2376.

**Yuan X, Xiong K, Li C Song Y. 2020a.** The complete mitochondrial genome of *Limassolla lingchuanensis* (Hemiptera: Cicadellidae: Typhlocybinae). *Mitochondrial DNA Part B* **5**(1), 229-230.

**Yuan X, Xiong K, Li C Song Y. 2020b.** Characterization of the complete mitochondrial genome of *Mitjaevia protuberanta* (Hemiptera: Cicadellidae: Typhlocybinae). *Mitochondrial DNA Part B* **5**(1), 601-602.

**Zhou N, Wang M, Cui L, Chen XX, Han BY. 2016.** Complete mitochondrial genome of *Empoasca vitis* (Hemiptera: Cicadellidae). *Mitochondrial DNA Part A*. **27**:1052-1053.

**Zhou XG, Cai WZ. 2017.** Mitochondrial phylogenomics of Hemiptera reveals adaptive innovations driving the diversification of true bugs. *Proc*. *Biol*. *Sci*. **284**:20171223.

**Zhao X, Liang AP. 2016.** Complete DNA sequence of the mitochondrial genome of the treehopper *Leptobelus gazella* (Membracoidea: Hemiptera). *Mitochondrial DNA Part A* **27**:3318-3319.

**Zhou X, Dietrich CH, Huang M. 2020.** Characterization of the Complete Mitochondrial Genomes of Two Species with Preliminary Investigation on Phylogenetic Status of Zyginellini (Hemiptera: Cicadellidae: Typhlocybinae). *Insects* **11**(10), E684.

**Table S3.** The best partitioning scheme selected by Partition Finder for different datasets.

**A.** The best partitioning scheme selected by Partition Finder for PCGs datasets.

| Subset | Best Model | Site (bp) | Partition names |
| --- | --- | --- | --- |
| 1 | GTR+I+G | 701 | cox3_pos1, cox2_pos1, atp8_pos1, atp6_pos1 |
| 2 | TVM+I+G | 1208 | cox2_pos2, cox3_pos2, cox1_pos2, atp8_pos2, atp6_pos2 |
| 3 | GTR+I+G | 2084 | nad6_pos3, nad3_pos3, cox2_pos3, cox1_pos3, atp8_pos3, cob_pos3, nad2_pos3, cox3_pos3, atp6_pos3 |
| 4 | GTR+I+G | 882 | cox1_pos1, cob_pos1 |
| 5 | GTR+I+G | 1269 | cob_pos2, nad3_pos2, nad4l_pos2, nad1_pos2, nad6_pos2, nad2_pos2 |
| 6 | GTR+I+G | 883 | nad1_pos1, nad5_pos1, nad4l_pos1 |
| 7 | TIM+I+G | 1264 | nad5_pos3, nad4l_pos3, nad1_pos3, nad4_pos3 |
| 8 | GTR+I+G | 882 | nad2_pos1, nad6_pos1, nad3_pos1, nad4_pos1 |
| 9 | GTR+I+G | 871 | nad5_pos2, nad4_pos2 |
|  |  |  |  |
|  |  |  |  |

**B**. The best partitioning scheme selected by Partition Finder for PCG12 datasets.

| Subset | Best Model | Site (bp) | Partition names |
| --- | --- | --- | --- |
| 1 | GTR+I+G | 701 | cox3_pos1, cox2_pos1, atp6_pos1, atp8_pos1 |
| 2 | TVM+I+G | 1553 | cox1_pos2, cox2_pos2, cob_pos2, atp6_pos2, cox3_pos2 |
| 3 | TVM+I+G | 531 | nad6_pos2, nad2_pos2, atp8_pos2, nad3_pos2 |
| 4 | GTR+I+G | 882 | cox1_pos1, cob_pos1 |
| 5 | GTR+I+G | 1765 | nad1_pos1, nad5_pos1, nad4l_pos1, nad2_pos1, nad6_pos1, nad3_pos1, nad4_pos1 |
| 6 | GTR+I+G | 1264 | nad5_pos2, nad1_pos2, nad4l_pos2, nad4_pos2 |

**C**. The best partitioning scheme selected by Partition Finder for PCG12R datasets.

| Subset | Best Model | Site (bp) | Partition names |
| --- | --- | --- | --- |
| 1 | GTR+I+G | 1553 | cob_pos1, atp6_pos1, cox1_pos1, cox3_pos1, cox2_pos1 |
| 2 | TVM+I+G | 1553 | cox2_pos2, cox1_pos2, cob_pos2, atp6_pos2, cox3_pos2 |
| 3 | GTR+I+G | 531 | nad6_pos1, nad2_pos1, nad3_pos1, atp8_pos1 |
| 4 | TVM+I+G | 531 | atp8_pos2, nad3_pos2, nad6_pos2, nad2_pos2 |
| 5 | GTR+I+G | 1264 | nad4l_pos1, nad5_pos1, nad1_pos1, nad4_pos1 |
| 6 | GTR+I+G | 1264 | nad5_pos2, nad1_pos2, nad4l_pos2, nad4_pos2 |
| 7 | GTR+I+G | 1752 | rrnL, rrnS |

**D**. The best partitioning scheme selected by Partition Finder for PCGR datasets.

| Subset | Best Model | Site (bp) | Partition names |
| --- | --- | --- | --- |
| 1 | GTR+I+G | 1558 | nad1_pos1, nad4l_pos1, nad5_pos1, nad3_pos1, atp8_pos3, atp6_pos1, nad4_pos1 |
| 2 | TVM+I+G | 1667 | atp8_pos2, nad3_pos2, cox3_pos2, atp6_pos2, nad2_pos2, nad6_pos2, cox1_pos2, cox2_pos2 |
| 3 | GTR+I+G | 1052 | nad6_pos3, nad3_pos3, atp6_pos3, cox3_pos3, cob_pos3 |
| 4 | GTR+I+G | 953 | nad5_pos2, nad4_pos2, nad4l_pos2, atp8_pos1 |
| 5 | GTR+I+G | 883 | cob_pos1, cox1_pos1 |
| 6 | GTR+I+G | 978 | nad2_pos3, cox1_pos3, cox2_pos3 |
| 7 | GTR+I+G | 852 | cox3_pos1, cox2_pos1, nad6_pos1, nad2_pos1 |
| 8 | GTR+I+G | 686 | nad1_pos2, cob_pos2 |
| 9 | K81UF+I+G | 1250 | nad4l_pos3, nad4_pos3, nad5_pos3, nad1_pos3 |
| 10 | GTR+I+G | 1802 | rrnS, rrnL |

**E**. The best partitioning scheme selected by Partition Finder for AA datasets.

| Subset | Best Model | Site (bp) | Partition names |
| --- | --- | --- | --- |
| 1 | MTART+I+G+F | 1679 | cox1, nad6, nad3, cox3, atp6, nad2, cox2 |
| 2 | MTART+I+G+F | 405 | atp8, cob |
| 3 | MTART+I+G+F | 1264 | nad4l, nad4, nad1, nad5 |

**Table S4.** Organization of five representative *Olidiana* species.

| Gene | Direction | Size (bp) | Start Condon | Stop Condon | Anticodon | Intergenic nucleotides |
| --- | --- | --- | --- | --- | --- | --- |
| *trnI* | J | 62 | - | - | GAT |  |
| *trnQ* | N | 67 | - | - | TTG | 1 |
| *trnM* | J | 66 (67) | - | - | CAT | −1 |
| *ND2* | J | 955 (954) | ATT | T/TAA | - | 0 |
| *trnW* | J | 62–66 | - | - | TCA | 0–−2 |
| *trnC* | N | 57–65 | - | - | GCA | −9–−15 |
| *trnY* | N | 63 | - | - | GTA | 0–−1 |
| *COI* | J | 1,536 | ATG | TAA | - | 2–10 |
| *trnL1*(UUR) | J | 66 (68) | - | - | TAA | 0 |
| *COII* | J | 676 | ATT/ATA | T | - | 0 |
| *trnK* | J | 71–73 | - | - | CTT | 0 |
| *trnD* | J | 63 (64) | - | - | GTC | −1 |
| *ATP8* | J | 150 | ATA/ATC | TAA | - | 0–1 |
| *ATP6* | J | 639 (642) | ATA | TAA | - | −1 |
| *COIII* | J | 778 | ATG | T | - | 0–1 |
| *trnG* | J | 60 (62) | - | - | TCC | 0 |
| *ND3* | J | 354 | ATT/ATA | TAG | - | 0 |
| *trnA* | J | 61 | - | - | TGC | −2 |
| *trnR* | J | 64–69 | - | - | TCG | −1–0 |
| *trnN* | J | 63–65 | - | - | GTT | −3–−2 |
| *trnS1* | J | 60–62 | - | - | GCT | −1 |
| *trnE* | J | 63(64) | - | - | TTC | −1–0 |
| *trnF* | N | 63–70 | - | - | GAA | −1–0 |
| *ND5* | N | 1,668–1,674 | ATT/ATC | TAA/TAG | - | −1 |
| *trnH* | N | 60–62 | - | - | GTG | 0 |
| *ND4* | N | 1,308 (1,311) | ATA/ATT | TAG/TAA | - | −1 |
| *ND4L* | N | 276 | ATG | TAA | - | −1 |
| *trnT* | J | 63 (64) | - | - | TGT | 1–2 |
| *trnP* | N | 61–62 | - | - | TGG | −1–0 |
| *ND6* | J | 477–483 | ATA/ATT | TAA | - | 1–8 |
| *CYTB* | J | 1,122 (1,134) | ATT/ATG | TAA/TAG | - | 4 |
| *trnS2*(UCN) | J | 64 (65) | - | - | TGA | −2–−1 |
| *ND1* | N | 939 | ATT/ATA | TAA | - | −7 |
| *trnL2*(CUN) | N | 66–68 | - | - | TAG | 0 |
| *rrnL* | N | 1,176–1,186 | - | - | - | 0 |
| *trnV* | N | 60–62 | - | - | TAC | 0 |
| *rrnS* | N | 729–788 | - | - |  | 0 |
| A+T- rich | J | 1,017–1,804 | - | - | - | 0 |

**Table S5.** Substitution saturation tests for the four datasets.

| Dataset | *Observed Iss* | *Iss.cSym*^1^ | *Psym*^2^ | *Iss.cAsym*^3^ | *Pasym*^4^ | Dataset | *Observed Iss* | *Iss.cSym*^1^ | *Psym*^2^ | *Iss.cAsym*^3^ |
| --- | --- | --- | --- | --- | --- | --- | --- | --- | --- | --- |
| PCG | 0.412 | 0.817 | 0.0000 | 0.571 | 0.0000 | PCGR | 0.415 | 0.818 | 0.0000 | 0.572 |
| PCG12 | 0.288 | 0.813 | 0.0000 | 0.570 | 0.0000 | PCG12R | 0.316 | 0.815 | 0.0000 | 0.571 |

^1^Critical values assuming a symmetrical tree.

^2^Signifcant difference between *Iss* and *Iss* and *Iss.cSym* (two-tailed test).

^3^Sym (two-tailed test). Critical values assuming an extreme asymmetrical tree.

^4^Signifcant difference between *Iss* and *Iss.cAsym* (two-tailed *t*-test).
